# From Matrices to Knowledge: Using Semantic Networks to Annotate the Connectome

**DOI:** 10.3389/fnana.2018.00111

**Published:** 2018-12-07

**Authors:** Sebastian J. Kopetzky, Markus Butz-Ostendorf

**Affiliations:** Biomax Informatics AG, Munich, Germany

**Keywords:** fMRI, DTI, gene expression, fkbp5, gmeb1, gmeb2, Human Connectome Project, Allen Human Brain Atlas

## Abstract

The connectome is regarded as the key to brain function in health and disease. Structural and functional neuroimaging enables us to measure brain connectivity in the living human brain. The field of connectomics describes the connectome as a mathematical graph with its connection strengths being represented by connectivity matrices. Graph theory algorithms are used to assess the integrity of the graph as a whole and to reveal brain network biomarkers for brain diseases; however, the faulty wiring of single connections or subnetworks as the structural correlate for neurological or mental diseases remains elusive. We describe a novel approach to represent the knowledge of human brain connectivity by a semantic network – a formalism frequently used in knowledge management to describe the semantic relations between objects. In our novel approach, objects are brain areas and connectivity is modeled as semantic relations among them. The semantic network turns the graph of the connectome into an explicit knowledge base about which brain areas are interconnected. Moreover, this approach can semantically enrich the measured connectivity of an individual subject by the semantic context from ontologies, brain atlases and molecular biological databases. Integrating all measurements and facts into one unified feature space enables cross-modal comparisons and analyses. We used a query mechanism for semantic networks to extract functional, structural and transcriptome networks. We found that in general higher structural and functional connectivity go along with a lower differential gene expression among connected brain areas; however, subcortical motor areas and limbic structures turned out to have a localized high differential gene expression while being strongly connected. In an additional explorative use case, we could show a localized high availability of fkbp5, gmeb1, and gmeb2 genes at a connection hub of temporo-limbic brain networks. Fkbp5 is known for having a role in stress-related psychiatric disorders, while gmeb1 and gmeb2 encode for modulator proteins of the glucocorticoid receptor, a key receptor in the hormonal stress system. Semantic networks tremendously ease working with multimodal neuroimaging and neurogenetics data and may reveal relevant coincidences between transcriptome and connectome networks.

## Introduction

The connectome – the entity of neural connections in the brain – is regarded as the key to understanding the human brain in normal functioning and disease (Seung, 2012; Shen et al., 2017; van Ooyen and Butz-Ostendorf, 2017). From the connectome, it can be inferred how intelligent someone is (Finn et al., 2015), how vulnerable the brain is to traumatic stress or neurodegeneration (Bigler, 2013; Crossley et al., 2014; McColgan et al., 2015; Contreras et al., 2015), or how well the brain will recover from damage (Kuceyeski et al., 2016). Connectomics is the modern approach to assess the connectome quantitatively (Sporns et al., 2005); the connectome is described as a mathematical graph (Power et al., 2011) with the nodes being the gray matter brain structures and the links of the graph being the white matter tracts connecting them. The weight of the link indicates the structural or functional connectivity between two brain structures. Graph theoretical measures – so-called complex network measures (Rubinov and Sporns, 2010) – quantify the integrity of the graph as a whole and can be used as biomarkers for neurological and psychiatric diseases (Smith, 2012; Drysdale et al., 2016; Woo et al., 2017). In addition to these global brain network parameters, diseases often can be attributed to pathological alterations in individual connections or specific sub-networks (Bartolomeo et al., 2007); this would require a more fine-grained description to understand their etiology.

The macroscopic functional and structural connectome – the connectivity between brain areas – can be measured non-invasively in human subjects. Different imaging techniques exist to measure functional brain connectivity, e.g., by functional magnetic resonance imaging (fMRI) (Kwong et al., 1992; Ogawa et al., 1992), by magnet encephalography (MEG) (Cohen, 1972) or even computed from standard electroencephalography (EEG) (Sakkalis, 2011). Functional brain connectivity is given in terms of correlation values between traces of electrical activity measured in different brain areas over time (Fox et al., 2005). Structural connectivity in terms of the thickness of anatomical fiber connections between brain areas can be measured by diffusion tensor imaging (DTI) in the living human brain (Basser et al., 1994). Additionally, structural connectivity can be measured by (immune-histochemical) fiber tracing (Oh et al., 2014 – in mice) or at high resolution by 3D polarized light imaging of post mortem brains (Axer et al., 2011, 2016). The relation between structural and functional connectivity, however, is highly complex (Hermundstad et al., 2013). Therefore, connectivity measurements need to be integrated from different modalities to obtain a most complete picture of the macroscopic connectome.

Standardizing processing workflows is a big issue in recent neuroimaging efforts to increase comparability of the results and interoperability among neuroimaging centers. *Semantic networks* are a formal approach, which is frequently used in neuroimaging to depict processing workflows. Essentially, a semantic network is a directed graph, which models the semantic relations between objects of a certain domain (Sowa, 1991). They have an important role in web technologies and especially for organizing Big Data in the life sciences (Losko and Heumann, 2017). For example, K-Surfer is a special extension of KNIME^®^ Analytics Platform^1^ for the management and analysis of MRI data (Sarica et al., 2014). Semantic networks can also be used to standardize and homogenize workflows by connecting them to established ontology terms and concepts, so-called provenance modeling. The NeuroImaging Data Model (NIDM) (Maumet et al., 2016) is an extension of the W3C PROV standard for the domain of human brain mapping. Semantic networks have also been used to enrich large clinical study data sets by semantic statistic vocabularies (Leroux and Lefort, 2015) and to achieve semantic interoperability of clinical research studies with an imaging component by the HL7 Fast Healthcare Interoperable Resource (FHIR) standard (Leroux and Raniga, 2017).

In the present study, we postulate a novel approach to semantically represent brain connectivity. Instead of describing the connectome as a collection of weights in a connectivity matrix, our approach uses a semantic network to describe each brain structure as an individual semantic object and the connections as the semantic relations among them. The novelty of this approach is that the semantic network creates a unified feature space in which all imaging modalities reporting connectivity can be compared and cross-modal analyses become possible. Moreover, by using semantic networks, connectivity can be put in a semantic context given by ontologies from structural or functional neuroanatomy (Brown et al., 2012) or pathology (Schriml et al., 2012). Furthermore, brain structures assessed by neuroimaging can be semantically enriched by available molecular biological data such as neural receptor densities (Zilles and Amunts, 2010) or gene expression in this brain area (Hawrylycz et al., 2012). Pathologic changes are very often specific indicators of brain diseases (Howes et al., 2015; Roy et al., 2016). Gene expression measurements of genes encoding for neural receptors in post-mortem brain tissue can be an additional indicator of the general availability of certain receptors in a particular brain area (Hawrylycz et al., 2012).

Individual receptor densities across the brain can be measured, e.g., with positron emission tomography (PET) (Wagner et al., 1983) or single photon emission photography (SPECT) (Eckelman et al., 1984) *in vivo*. Although they show characteristic patterns in healthy brains – so-called neural receptor finger prints (Zilles and Amunts, 2010), changes in receptor densities are reversible and are mostly a rather volatile marker of brain diseases (Volkow et al., 2011). Alterations in structural connectivity are much slower and much longer-lasting than molecular changes in receptor densities and functional connectivity, and are therefore often a much better predictor of the actual disease state (Contreras et al., 2015). Due to activity-dependent structural plasticity (Butz et al., 2009; Butz-Ostendorf and van Ooyen, 2017) there is a complex reciprocal interaction between neural transmitter receptors, functional connectivity, and structural connectivity rewiring. Longitudinal neuroimaging of receptor densities together with functional and structural connectivity in the individual patient is therefore essential for a complete picture of the disease development and the effect of treatment.

In this work, we give a detailed introduction to the creation of a semantic network model of the connectome. We describe how to query functional and structural connectivity from the USC Multimodal Connectivity Database (Brown et al., 2012; Brown and van Horn, 2016) contributing to the Human Connectome Project (Bardin, 2012) and gene expression data from the Allen Human Brain Atlas (Hawrylycz et al., 2012) to create joined structural, functional connectivity and transcription networks from healthy human brains. We also queried the spatial gene expression distribution of individual genes and co-localized them with functional brain networks. Furthermore, we show how to use anatomical and functional brain ontologies such as the “neuroanatomical domain of the foundational model of anatomy ontology” (FMA) (Nichols et al., 2014) or the ontologies provided by the Brede database for functional imaging (Nielsen, 2003) to annotate the human connectome with neuroanatomical meta-information.

## Methods

### The Brain Science Knowledge Model at a Glance

While the aforementioned applications of semantic networks in neuroimaging focus on procedural aspects of neuroimaging, we here propose a novel declarative approach to explicitly represent knowledge contained in multi-modal brain data by deploying semantic networks. Semantic networks in our approach describe the semantic relations of data items from very heterogeneous database sources. The nodes of the semantic network graph, the semantic objects, represent available data and knowledge from the field of neuroscience. To accommodate different kinds of data, there are several semantic objects kinds. These are static template objects, from which new dynamic object types can be dynamically derived. These static object kinds are “element,” “context,” “experiment,” “ontology,” and “annotation.” For example, the semantic object type “patient” could be dynamically derived from the static semantic object kind “element,” while “fMRI,” which holds the values of a functional connectivity matrix (Brown and van Horn, 2016) would be derived from “experiment,” or “FMA” (The Foundational Model of Anatomy, cf. Rosse and Mejino, 2003; Nichols et al., 2014) would be an object of kind “ontology.” Once the semantic network is set up, instances of those semantic objects are created by importing individual data sets. Data import is described in the section “The underlying technology.”

The directed links of the graph are semantic relations that are defined by a feed-forward definition and a backward definition. For example, a patient “is assessed by” an fMRI, and an fMRI “assesses” a patient. For each patient assessed by fMRI, an instance of this semantic relation is created upon data import. Furthermore, annotations can be added to any semantic object or relation to add meta-information. In particular, annotations are needed to store patient data or to maintain the versioned protocol of an fMRI measurement, for example.

**FIGURE 1:**
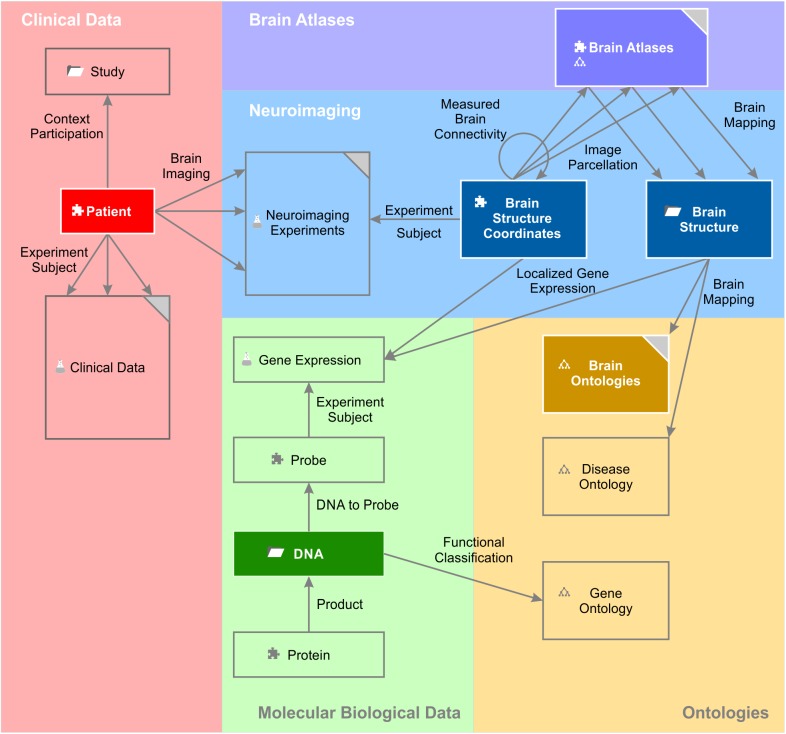
A simplified brain-data knowledge model. The knowledge model can be subdivided in at least five sub-domains: clinical, neuroimaging, molecular biological data, brain atlas, and brain ontology. Rectangles indicate semantic object types or groups of corresponding semantic object types indicated by the gray triangle in the top right corner. Darker background colors of rectangles indicate key objects of the subdomains. Semantic object types are derived from a static set of object kinds indicated by a small icon preceding the object name. The puzzle tile icon indicates the most basic object kind the “element.” The folder icon indicates the object kind “context,” the glass flask icon the object kind “experiment” and the tree diagram icon the object kind “ontology.” The different “brain atlases” can be derived either from kind element or ontology. The arrows indicate the semantic relations between the different objects or even between the same object types (e.g., “measured brain connectivity”). Classes of semantic relations can connect different multiple source and target objects with each other. For example, the relation class “brain imaging” relates the patient with data from the different imaging modalities. “Experiment subject” is a special relation belonging to the semantic object kind experiment. Every experiment has got exactly one semantic object type as its subject. It identifies the individual data entries of this experiment (cf. main text).

The entity of all semantic objects and relations constitute the knowledge model of a domain – here the field of brain science (Figure 1). The knowledge model comprises semantic objects for all available clinical data, neuroimaging data sets, molecular biological databases as well as brain atlases and brain ontologies. It is not fixed, but can be dynamically extended by new semantic objects. Very much like putting Lego^TM^ bricks onto each other, new semantic relations can be created with existing objects independently of the underlying database specifications.

**FIGURE 2:**
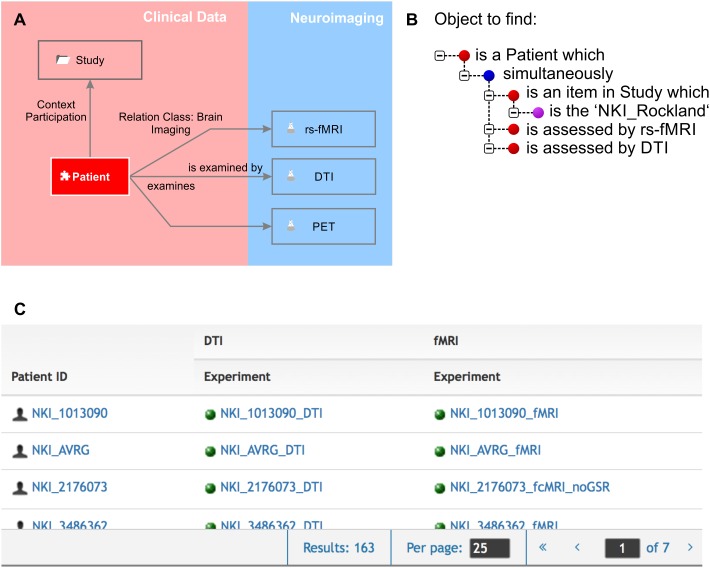
Knowledge model and query language. **(A)** We zoomed in to the clinical data and the neuroimaging sub-domains. The Relation Class “brain imaging,” indicated by the gray arrows from “patient” to the different imaging experiments, is defined by “is examined by” and “examines” in forward and backward directions, respectively. The “patient” participates in the context “study.” **(B)** The query language uses the object names and the definitions of the relations. Red bullet points indicate semantic objects, purple bullet points labels and identifiers, and blue bullet points logical operators. **(C)** The result of the query shown in **(B)** is a list of patients that fulfill the required conditions that they are part of the NKI Rockland study (Nooner et al., 2012) and have rs-fMRI and DTI experiments available.

Knowledge is made explicit by querying the knowledge model. One would query the knowledge model by using the natural language terms defined by the semantic object type names and by the relation definitions. A simple query would be as follows: The object to find is an element “patient” which “is assessed by” an experiment “fMRI.” The result would be an availability table containing all patients who underwent any fMRI routine. The knowledge model can be very specific as resting state fMRI can be separately represented from any particular task-dependent fMRI protocol giving a precise overview of which assessments were performed for which patient. A second query could, for example, report all brain structures which strongly express a certain gene and which are involved in a certain brain disease. Queries can ask for any combination of directly or remotely related semantic objects by following a path through the semantic network (Figure 2).

### The Underlying Technology

The semantic networks of the knowledge model were set up in the BioXM^TM^ Knowledge Management Environment version 6.1 by Biomax Informatics AG, Planegg, Germany. The BioXM platform mediates the database access for every semantic object (Losko et al., 2006; Losko and Heumann, 2009; Maier et al., 2011; Losko and Heumann, 2009). Every instance of a semantic object corresponds to a database entry. Once a knowledge model is defined, data can be uploaded to the underlying databases. Import operations use the same semantics as defined by the knowledge model so that the actual database routines for data import are encapsulated by the platform. With every newly imported data item, a new instance of the respective semantic object is created. In this way, the knowledge model becomes enriched by data. The built-in query language can be used to query any data that matches the query criteria. The data retrieval from the database is again organized by the platform. The platform technology creates a unified data space irrespective of the different data sources and the underlying database technology and enables working on a highly abstract level using the semantic relations in the knowledge model.

**FIGURE 3:**
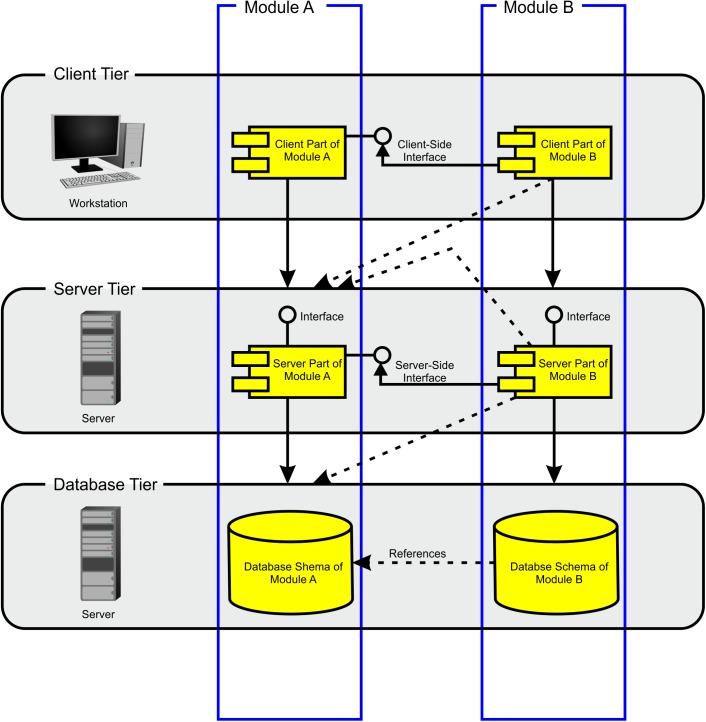
Modules architecture. The BioXM platform consists of three tiers: client, server, and database. The database tier contains all the database schemes. The user does not directly access databases, but works through a Java^®^ client that enables setting up the knowledge model, import data and to query the knowledge model. The server tier mediates between the user and the database tier. There is a module which defines the functionality for each object kind from user input to database access. There are interfaces between modules on the client and the server tiers (solid lines). Modules are cross-referenced on the database tier and between different tiers (dashed lines) enabling cross-referencing and querying multiple data bases.

The BioXM platform is based on Java^TM^ client-server architecture (Losko and Heumann, 2017). The server supports MySQL^®^ or Oracle^®^ relational database management systems (RDBMS). Data can be imported directly through a Java-based BioXM client application, web portal, command-line interface or any other application accessing the BioXM application programming interfaces (APIs). The BioXM platform technology consists of three tiers (Figure 3), a client tier, a server tier and a database tier. The BioXM platform implements the functionality to operate across the different tiers in modules. A module, e.g., for a semantic object or a relation provides generic graphical user interface (GUI) components for working with objects and relations at client-tier level, server communication routines at server-tier level, and the required database schemes at database-tier level to store and retrieve instances of the semantic objects and relations in and from the database. Moreover, the module contains a framework for audit object properties and the XML export of object lists. The use of the BioXM platform technology eases working with semantic networks tremendously; however, the concept of annotating connectome data sets by semantic networks is not at all limited to the usage of the BioXM platform. Any other tools for creating semantic networks and managing the database access can be used as well.

### Detailed Description of the Brain Science Knowledge Model

The knowledge model can be subdivided into at least five different sub-domains: clinical, neuroimaging, molecular biological data, brain atlases, and brain ontologies. In addition, the knowledge model can contain sub-networks that describe processing workflows for every neuroimaging or molecular biological method. Sub-domains and sub-networks are not disjunctive but to a certain degree overlapping and interlinked concepts. They help structure the whole knowledge model. Every sub-domain has central semantic objects to which newly added objects can be easily linked. The semantic relations between the central objects of the subdomains form the backbone of the knowledge model.

### A Patient-Centric Knowledge Model

**FIGURE 4:**
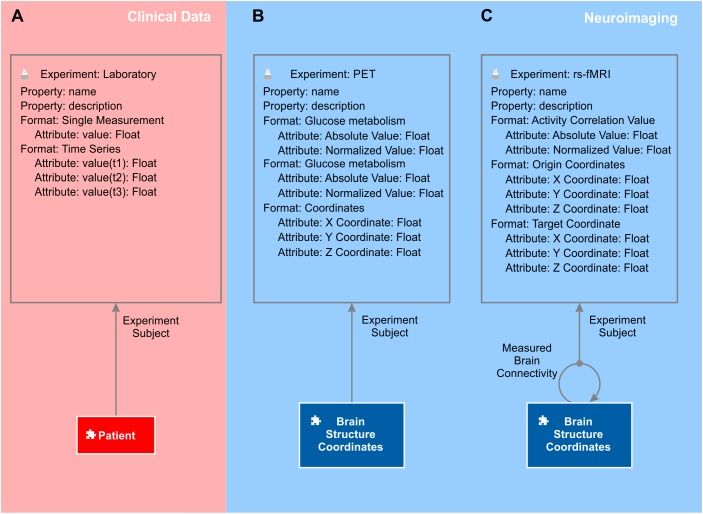
Laboratory and neuroimaging experiments. Every experiment is defined by its name, method, subject, and format. Every format can contain different attributes to accommodate the data. **(A)** In the clinical subdomain, the experiment method “laboratory” contains all measurements of the patient such as blood values. The patient is the subject of each experiment method laboratory. The main differences between laboratory experiments and neuroimaging experiments is that the latter do not have the patient as the experiment subject. **(B)** Neuroimaging experiments that measure, for example, glucose metabolism at a particular location in the brain, such as the experiment method “PET,” have the element “brain structure coordinates” as the subject. **(C)** Experiments that measure connectivity between “brain structure coordinates” (e.g., by experiment method “rs-fMRI”) have the semantic relation “measured brain connectivity” as the subject.

The ultimate goal of the present approach is to integrate all data of the individual patient with the available knowledge in the scientific field. The element “patient” is therefore the central semantic object of the clinical sub-domain. The patient object is annotated with all required patient information containing a unique patient ID, patient address and health security status, etc. All processed neuroimaging data are linked to the patient via the semantic relation “brain imaging.” The relation is defined by “is assessed by” and “assesses” in forward and backward directions, respectively. Further, the patient is an item in the context “study.” The object kind “context” is used to group semantic objects. Instances of the semantic object “patient,” the individual patients, can be dynamically added to a context “study” to organize existing or plan future studies. The patient is also subject of the experiment “laboratory” (Figure 4A) and relates to all neuroimaging experiments. Experiments are used to store and organize large sets of (e.g., numeric) data. Experiments are defined by their method, their format and their subject. The experiment method “laboratory” contains all experiments with measured lab values of patients. One experimental data entry of that experiment has a subject, which, in this case, is one instance of the semantic object patient and contains exactly one measurement of the specified method and format of this patient (Figure 4).

### Combining Declarative and Procedural Aspects in the Knowledge Model

For reproducibility and interoperability of neuroimaging, it is crucial that the documentation of the used processing workflows is stored with the obtained results. Approaches to define workflows, e.g., KNIME^®^ workflows^1^, so far do not offer the possibility to organize the results together with the workflows. The knowledge model, which we are introducing here, combines declarative knowledge with procedural knowlegde; it contains semantic objects that represent data as well as those that represent processing, analysis or assessment tools. The knowledge model contains, e.g., specialized contexts and elements for raw data types such as “Nifti Image” and for each processing task such as “BET” (brain extraction tool of the FSL tools library^2^), “FLIRT” or “DEFACE T1” to name a few. Outputs of these processing steps are again represented by respective elements, for example, the “T1_brain” or “T1_brain_mask” and many more. The different objects are connected via semantic relations according to the chosen workflow. All semantic objects and relations can be annotated by the relevant meta-information of the processing task. For example, the element BET has an associated annotation “BET parameters” that contains the chosen parameters and their ranges and default values as an example configuration, i.e., fractional intensity threshold (0–>1); default = 0.5; <g> vertical gradient in fractional intensity threshold (-1– >1); default = 0; <r> head radius (mm not voxels), initial surface sphere is set to half of this; <c> <x y z> center-of-gravity (voxels not mm) of initial mesh surface; and <t> applied thresholding to segmented brain image and mask (see FSL documentation for detailed explanation of the parameters^3^). An example configuration of the T1 processing pipeline in accordance to Alfaro-Almagro et al. (2018) is shown in Supplementary Figure S1.

The semantic subnetwork representing a processing workflow is connected to the element patient. At the moment we start a neuroimaging workflow, we instantiate the knowledge model by creating an instance of the context Nifti image and a semantic relation from element patient to Nifti image. With each state transition in this processs, we create a further related object and parameter annotations representing the performed task. By doing so, we automatically document the full processing workflow. Moreover, as part of the knowledge model, the entire documentation is searchable by the built-in query mechanism. We can, for example, ask for all patients who were assessed by a certain workflow and particular parameter settings. Comparisons between results of patients with parameter settings A and parameter settings B become easily possible.

### Representing the Connectome in the Knowledge Model

To accommodate any processed neuroimaging data (brain connectivity, receptor density values, brain volume and cortical thickness, etc.), we introduced an element “measured brain coordinates.” The intention was that every coordinate in an individual or ideally in a standardized brain at which a measurement was taken becomes an individual semantic object to which other semantic objects can relate. Mapping all coordinates of an individual patient to a standardized brain like the MNI-152 (Mazziotta et al., 1995) poses the great advantage that knowledge of all patients can be accumulated in the knowledge model. The element measured brain coordinates is the key object that turns the graph of a (macroscopic) connectome network into a semantic network as we can now introduce any type of functional or structural connection as a semantic relation between two measured brain coordinates objects (Figures 4B,C).

Whenever a structural or functional connection between two 3D locations in the brain is measured an instance of the semantic relation between the corresponding measured brain coordinates objects is created (Figure 4C). The relation is defined as “connects to” and “is connected from” for the forward and the backward definitions, respectively. Because semantic relations are always directed, it is possible to accommodate directed connectome information even though neuroimaging data sets are often undirected. It must be ensured that relations are only created between coordinates belonging to the same coordinate frame of an individual or a standardized brain. The more neuroimaging data sets reporting connectivity of individual brains are imported to the semantic network, the more measured brain connectivity relations are created. Ultimately, the semantic network accumulates the knowledge of brain connectivity that becomes transcendent over the single neuroimaging measurements.

### Integrating Functional and Structural Brain Connectivity

Functional and structural brain connectivity measured, e.g., by resting state fMRI (rs-fMRI) or DTI, respectively, is usually stored in *nxn* matrices, so-called connectivity matrices, with *n* being the number of individual brain areas. The approach works accordingly for every fMRI paradigm reporting connectivity. The values at each matrix entry range from -1 to 1 for functional connectivity indicating activity correlations over time, and are positive integer or float values for structural connectivity indicating fiber densities or probabilities, respectively. We integrated connectivity matrices into semantic networks by using the semantic object kind experiment and created an experiment method “rs-fMRI” and a method “DTI.” Further experiment methods, e.g., EEG and MEG can be added easily as needed. One experiment contains all connectivity values of an individual patient; however, the subject of this experiment is not the semantic object patient as for laboratory experiments, but the semantic relation measured brain connectivity (Figure 4C). One particular experimental data entry therefore contains the strength of a certain functional or structural connection in one neuroimaging assessment of an individual patient. The experiment format to capture connection strengths chosen is “absolute correlation value” and “normalized correlation value” for “fMRI,” and “absolute fiber density,” and “normalized fiber density” for deterministic DTI, and “probability” for probabilistic fiber tracking in a DTI data set.

### Representing Localized Brain Data Such as Cortical Thickness and Receptor Densities

In addition to brain connectivity data, we can integrate measurements at a certain 3D image location like cortical volume, glucose metabolism or receptor densities. For this, we defined experiment methods “cortical volume” and “PET” with the measured brain coordinates object as the subject (Figure 4B). Each experiment method PET can contain all measurements from a PET scanner. [^18^F]fluoro-deoxyglucose ([^18^F]FDG) is a radiopharmaceutical to monitor changes in glucose metabolism and, hence, brain activity. Cortical volume together with glucose metabolism of individual brain regions such as hippocampus, amygdala, and entorhinal cortex may indicate different forms of dementia (Del Sole et al., 2016). Further radioligands that bind to individual neurotransmitter receptors exist (Eckelman et al., 1984; Steiniger et al., 2008; Roy et al., 2016). The experiment method “PET” therefore contains different experiment formats such as “18FDG” or “receptor density” for measuring glucose metabolism and receptor density measurements, respectively. Each experiment “PET” contains all PET measurements of one individual patient. Each experimental data entry of this experiment in turn contains all values measured at a specific 3D location in the patient’s brain.

### The Knowledge Model Enables the Parallel Use of Multiple Brain Atlases

**FIGURE 5:**
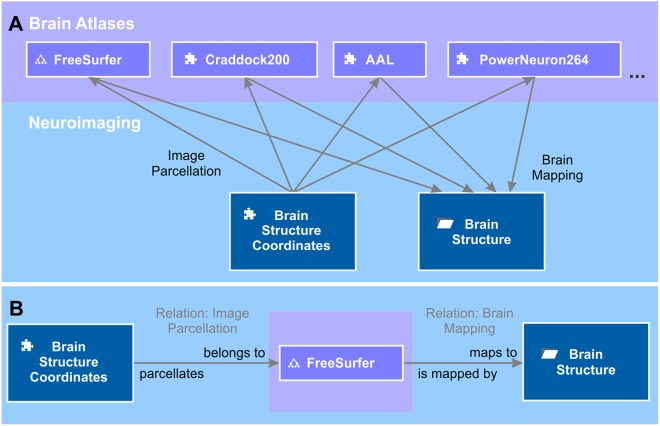
Brain mappings. **(A)** Brain atlases are used to parcellate the brain image into volumes that correspond to a certain brain area. The relation between volumes and brain areas are defined by the brain atlas. The tree diagram icon and the puzzle icon indicate whether the brain atlas is an ontology or an element. Entries of the different brain atlases are mapped to a unified repository of brain structures – the context “brain structure.” The semantic object kind “context” provides the possibility of grouping semantic objects that are of relevance for this brain structure, e.g., literature references (not shown). **(B)** The semantic relation “image parcellation” that relates the element “brain structure coordinates” to a brain atlas, e.g., “FreeSurfer” is defined by “belongs to” and “parcellates” in forward and backward directions. Likewise, the relation “brain mapping” is defined by “maps to” and “is mapped by.”

The brain area or structure to which a voxel at a certain 3D localization in a brain image data set belongs depends on the brain atlas chosen to parcellate the brain image. The same 3D coordinates can, at the same time, belong to a large brain structure like “brain stem” or, if a high-resolution atlas was used, to a smaller structure like “midbrain” or even “substantia nigra.” Therefore, we kept the representation of 3D localizations separate from the representation of brain structures in the semantic network. To link between the two, we introduced a semantic relation “image parcellation” (Figure 5), which we defined by the forward definition “belongs to” and the backward definition “parcellates.” The source of the relation is the object measured brain coordinates and target is a brain atlas entry. The brain atlas is either an element or an ontology depending on whether the brain structure listing comes as a flat listing or as a hierarchically organized ontology. Such brain structure listings are, for instance, the Automated Anatomical Labeling (AAL) (Tzourio-Mazoyer et al., 2002), Talairach atlas (Talairach and Tournoux, 1988), Craddock200 (Craddock et al., 2012), PowerNeuron264 (Power et al., 2011), FreeSurfer (Reuter et al., 2012^4^) and many more. We created elements “AAL,” “Taliarach,” “Craddock200,” “PowerNeuron264,” and an ontology “FreeSurfer.” The list of brain atlases for image parcellation is constantly growing. Further semantic objects for new brain atlases can be added at any time. Brain atlases for imaging parcellation provide tables that relate a set of measured 3D coordinates referring to a standardized brain, e.g., the MNI-152 brain (Mazziotta et al., 1995) to a specified and labeled brain structure. Based on these tables, we created instances of the semantic relation image parcellation from measured brain coordinates to, e.g., AAL, Craddock200 and FreeSurfer to name a few.

### Mappings Between Brain Atlases in the Knowledge Model

One challenge of the co-existence of multiple brain atlases is the heterogeneity of terms used to label corresponding brain structures across different atlases. The great variety of different terms used for the same brain structure is a big hurdle for semantic data integration. Moreover, looking from a 3D image perspective, the parcellated volume that is referred to by any two corresponding brain structure labels of different atlases is not necessarily identical. Meaning that the terms of different atlases cannot be safely treated as synonyms, but require manual evaluation in each case. The intention of semantic integration of brain data for knowledge management is to condense dispersed information into unified semantic objects. What we can achieve for multimodal brain imaging and neuroanatomical data is to join information about, similarly, defined brain structures. To accumulate this information we created a semantic object “brain structure” that relates to all brain atlases and ontologies, but is, *per se*, an independent repository of representative brain structures (Figure 5). The relation class “brain mapping” contains all semantic relations which connect related brain structures in different brain atlases. It is defined by the forward definition “maps to” and the backward definition “is mapped by.” A relation class contains all semantic relations with identical definitions but with multiple sources and targets. Due to the aforementioned concern, the mapping will never be perfect, but must be understood as a “best match” (Supplementary Table S1).

### A Generalized Knowledge Base for Brain Structures

**FIGURE 6:**
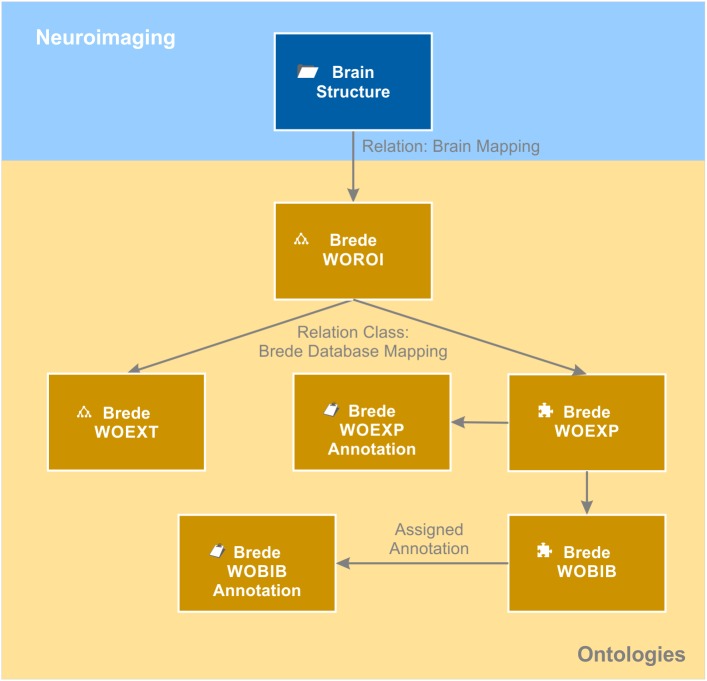
Brain ontologies. Brain ontologies form a further sub-domain of the brain data knowledge model. The “brain structure” object that contains a repository of known brain structures is matched to structural and functional brain ontologies. The example shows a representation of the Brede database (Nielsen, 2003). The database consists of an ontology of brain structures (WOROI), an ontology of external components (WOEXT), and a list of cognitive experiments (WOEXP) that are further related to a list of literature references (WOBIB). Annotation objects indicated by the clipboard icon hold additional information such as the name of the cognitive tests (WOEXP annotation) or the PubMed ID of the cited paper and other meta-information (WOBIB annotation). Ontologies are indicated by the tree diagram icon while flat lists stored by the semantic object kind “element” are indicated by the puzzle icon.

The knowledge model brings together data from an individual patient with generalizable knowledge about the brain. In order to avoid replicating general knowledge for every patient, we introduced the context “brain structure.” It acts as a hub in the semantic network as it relates to all measured brain coordinates from the individual patients as well as to all ontologies storing meta-information about brain structures. Available information about a certain brain structure from functional and structural neuroanatomy, molecular biology and neuroimaging of individual patients is directly or indirectly linked to the matching brain structure object. We used the Allen Human Brain Atlas ontology (Hawrylycz et al., 2012) as the naming convention for our brain structure repository. This enabled us to directly link every brain structure object to its corresponding entry in the “Allen Human Brain Atlas” ontology. As a result, each brain structure becomes automatically categorized anatomically by this atlas ontology. By relating the brain structure object also to the neuroanatomical domain of the Foundational Model of Anatomy ontology (FMA) (Nichols et al., 2014), additional anatomical information is provided. In addition to the usual “is a” relation, the FMA ontology provides anatomical and topological relations such as “is adjacent to,” “provides input to,” “receives input from” and many more. By relating every brain structure to the corresponding entries in the FMA ontology, this anatomical knowledge becomes integrated in the knowledge model and globally available, e.g., for annotating the connectome of the individual patient. Moreover, each brain structure is related to functional anatomical knowledge. We mapped the Brede WOROI ontology (Nielsen, 2003) to the matching brain structure object. Brede WOROI contains a hierarchically organized list of brain structures, which are, in turn, related to an ontology of cognitive functions called Brede WOEXT. Finally, mappings between Brede WOEXT and Brede WOEXP, a further Brede ontology containing cognition experiments, exist. We integrated these three ontologies, included the provided mappings between them and integrated them into the knowledge model (Figure 6). Thereby, it becomes possible to query all brain structures that are related to a certain cognitive function or were tested in a cognitive experiment.

### Semantic Enrichment of Brain Data With Molecular Biological Data

**FIGURE 7:**
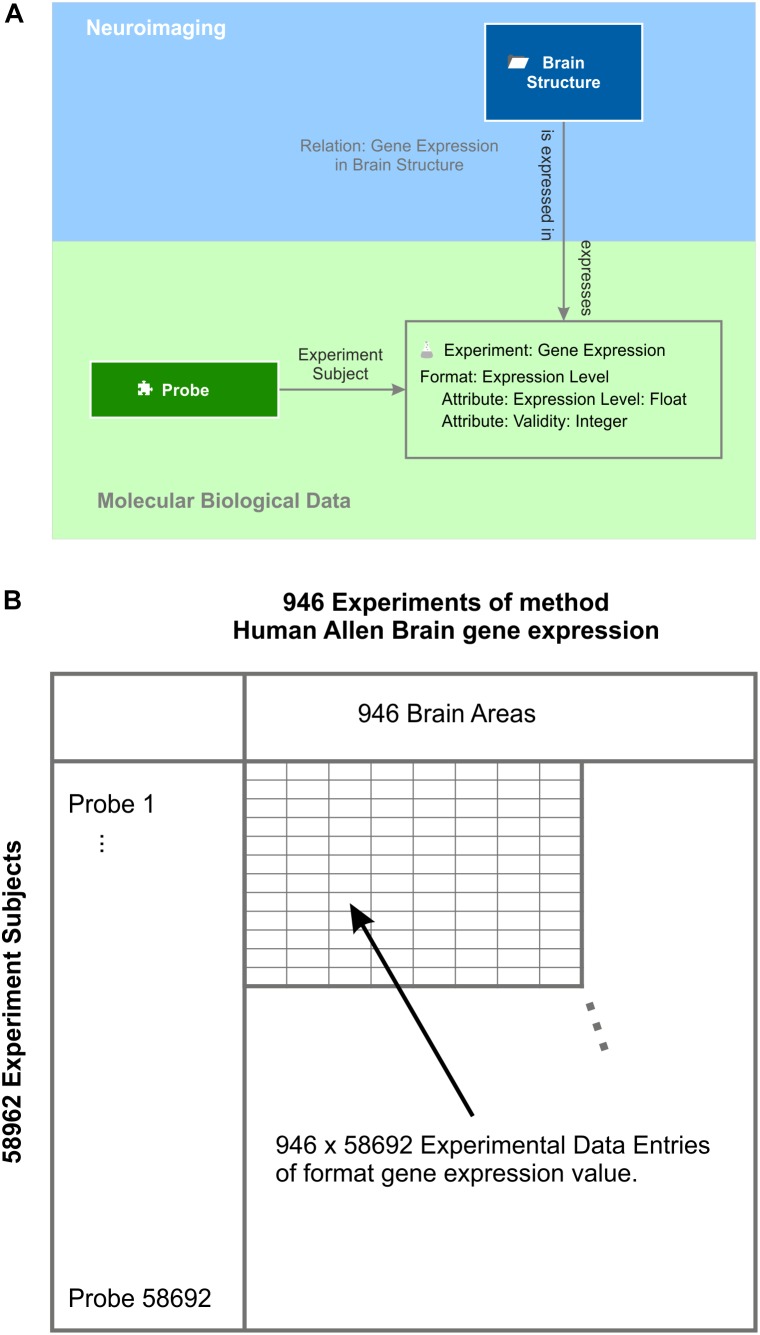
Gene expression experiments. **(A)** Experiments of method “gene expression” have the expression level format. They contain the actual value and a validity signal provided by the Allen Human Brain Atlas (Hawrylycz et al., 2012). Each experiment contains the gene expression values measured by all probes and relates to the respective “brain structure” element by the semantic relation “gene expression in brain structure.” The probe is the subject of each gene expression experiment. **(B)** Subject and experiment name expand a table with the subjects being the row labels and the experiment names being the column labels. In this example, the probes label the rows and the brain areas the columns. This table holds all data that is stored by the experiment method gene expression – in this case, 946 × 58692 experimental data entries taken from one post-mortem brain (Hawrylycz et al., 2012).

As an example for molecular biological data localized in the brain, the Allen Brain Institute provides a comprehensive multi-array gene expression data set of six human post-mortem brains (Hawrylycz et al., 2012). The expression of every human gene was measured by applying at least two probes per gene. All measurements are labeled by the MNI 152 coordinates (Mazziotta et al., 1995) from which they were taken and mapped to the Allen Human Brain Atlas. A semantic object experiment method “Allen Human Brain Atlas gene expression” integrates expression levels into the knowledge model (Figure 7). Every experiment of this method is identified by an identifier of the donor and the location at which the gene expression was measured. Each experiment contains the expression values measured by about 60 thousand probes. One experimental data entry of the experiment is identified via the experiment’s subject, which is the element “probe.” The element probe is semantically related to the context “DNA” via the relation “DNA to probe,” which is defined as follows: a DNA “is detected by” a probe and a probe “binds to” a DNA. Via the intermediate semantic object probe, the gene expression value becomes related to a particular gene for which functional classification is available through the Gene Ontology (Ashburner et al., 2000; The Gene Ontology Consortium, 2017).

## Exploratory Use Cases

### Working With Functional Brain Networks

Now that the knowledge model for brain data is set up and enriched with data, we can use the BioXM query language (Losko and Heumann, 2017) to work with the knowledge model and assemble data from any desired integrated source. For example, we can use the knowledge model to create templates of functional brain networks (FBNs) assisted by the knowledge from brain atlases and ontologies. The FBN template can be used to filter an individual connectome data set or differences between data sets. The so-filtered connectome data set enables the clinician to observe the status-quo or changes in the brain of the patient. For example, patients with a “dysconnection” syndrome are known to have a faulty wiring of limbic connections associated with hallucination, delusions or neglect (Bartolomeo et al., 2007). Schizophrenia is considered to resemble a dysconnection syndrome (Weinberger and Lipska, 1995; Bagorda et al., 2006). It now becomes possible to easily relate behavior to quantitative changes in, for example, this particular brain connectivity.

**FIGURE 8:**
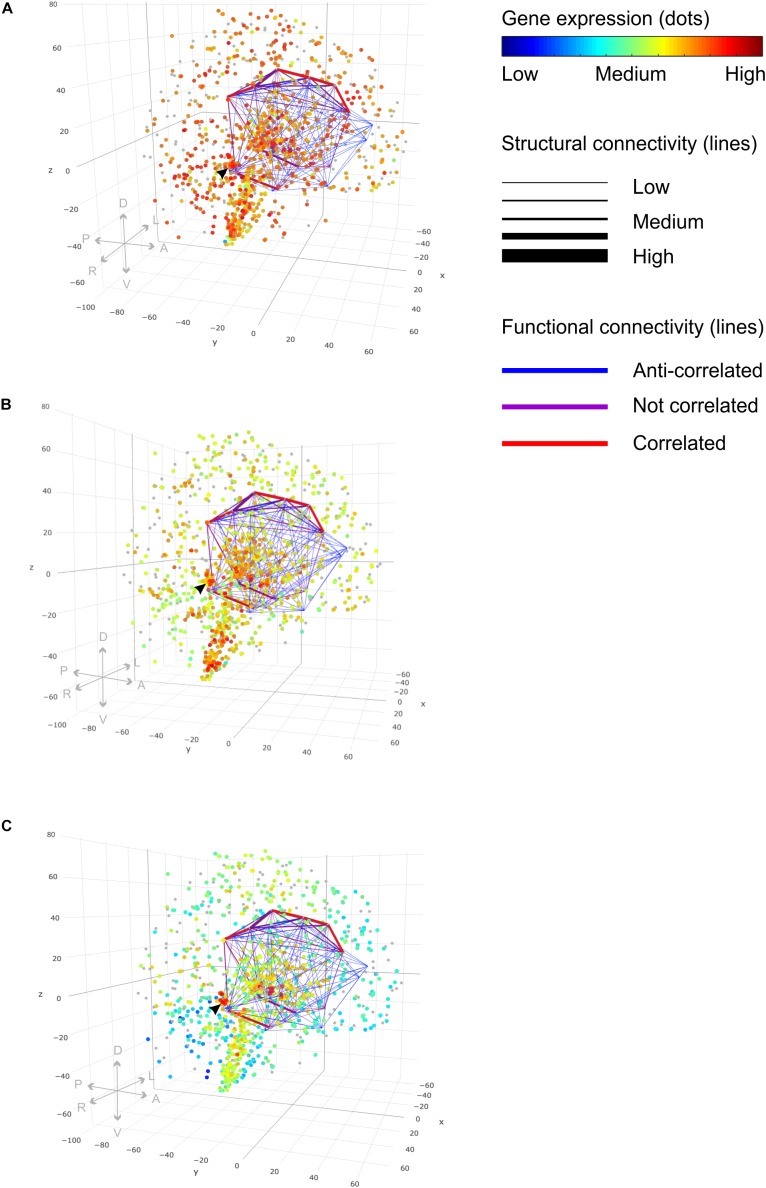
Functional brain networks and gene expression. 3D lattice graphs in all three panels show connectome information on structural (line strength) and functional connectivity (line color) of the limbic lobe. Red lines indicate strong functional connectivity and blue lines weak functional connectivity. Strong functional connectivity means that activity of connected brain areas is correlated in time. Weak functional connectivity means that brain areas’ activities are anti-correlated. The black arrow head indicates the location of the hippocampal formation — a connection hub of the limbic lobe sub-network. Data were taken from rs-fMRI and DTI connectivity matrices from the NKI Rockland study (Nooner et al., 2012) through the USC Multimodal Connectivity database (Brown and van Horn, 2016). Legend on directions in the plot: anterior, “A”; posterior, “P”; left, “L”; right, “R”; dorsal, “D”; ventral, “V.” **(A)** Gene expression values for the gene “gmeb1” encode for the “glucocorticoid modulatory element-binding protein 1.” We see ubiquitously high gene expression with a particular accumulation of high values in the vicinity of the hippocampal connection hub region indicated by the black arrow. **(B)** Gene Expression for the gene “gmeb2” encoding for the “glucocorticoid modulatory element-binding protein 2” shows accumulation of high gene expression in the hippocampal formation, too. **(C)** Gene expression of gene “fkbp5” involved in receptor binding regulation of the glucocorticoid receptor shows highly localized increased values in the hippocampal formation, too. (See the main text for explanation.) Different gene expression values of multiple probes binding at the same location were plotted with 50% transparency to display the results of all probes. Gene expression values were taken from the Allen Human Brain Atlas (Hawrylycz et al., 2012).

The query to filter a connectome by a FBN template could look like the following: “Find all measured brain coordinates which ‘belong to’ a Craddock200 which ‘maps to’ a brain structure which ‘maps to’ an Allen Human Brain Atlas ontology entry which ‘is inferred from’ an Allen Human Brain Atlas ontology entry which ‘is the’ 4219 limbic lobe.” The query makes use of the ontology relation ‘is a’. It returns a table of the origin and the target brain structure with the corresponding measured brain coordinates, the fiber density and the activity correlation values. These results can be used to create a lattice graph in the MNI coordinate frame (Figure 8). Line thickness indicates structural connectivity and line color functional connectivity. We used connectome data sets from the NKI Rockland study (Nooner et al., 2012) available through the USC Multimodal Connectivity Database (Brown et al., 2012, 2016), part of the Human Connectome Project (Bardin, 2012). Each data set contained connectivity matrices for functional and structural connectivity derived from fMRI and DTI raw data images, respectively. The Craddock200 brain atlas (Craddock et al., 2012) was used for this study to parcellate brains into 188 different 3D coordinates belonging to 126 different anatomical areas. Applying the FBN limbic lobe template to an averaged DTI connectivity data set “nki_dti_avg” and an averaged rs-fMRI connectivity data set “nki_fc_avg” from the NKI Rockland study (Nooner et al., 2012) revealed 110 pairs of structural and functional connections of the limbic lobe (Figure 8 and Supplementary Table S2). It has been averaged over 196 individuals ranging fom age 6 to 89. The two data sets were obtained from the USC Multimodal Connectivity database (Brown et al., 2012).

### Combining Connectome With Molecular Biological Data

The knowledge model enables us to query the data from any perspective. In the aforementioned example, we queried the data from the neuroimaging perspective and obtained measured connection strengths indicating structural and functional connectivity of an individual subject. In the next example, we approach the data from a molecular biology perspective. The query could ask for brain structures which relate to an Allen Human Brain gene expression experiment for donor9861 that has an experiment subject, which is a probe which binds to a DNA which is fkbp5. FK506 binding protein 51, or short fkbp5, is involved in the regulation of glucocorticoid receptor sensitivity and is part of the stress hormone regulation system (Binder, 2009). Polymorphisms in the gene encoding for fkbp5, which can lead to a dysregulated stress response, might be a risk factor for stress-related psychiatric disorders. The query returned 211 combinations of probes, which bind to fkbp5, and MNI 152 coordinates in the donor brain 9861 with gene expression values greater than 8 and with a signal significantly greater than background noise (PACall value equal to 1) (Supplementary Table S3). The strongest gene expression for fkbp5 was seen in the hippocampal formation (Figure 8).

In a second query, we searched for the two genes “gmeb1” and “gmeb2” for the glucocorticoid modulatory element binding proteins 1 and 2, respectively. The query returned 79 localized probes in the brain, which bind to gmeb1 (Supplementary Table S4), and 11 probes (Supplementary Table S5), which bind to gmeb2, with gene expression values greater than 8 and with a signal significantly greater than background noise (PACall value equal to 1). We wanted to know whether those modulatory elements of the glutamate receptor are co-localized with fkbp5, which may suggest that polymorphisms of these genes have a putative role in mental stress tolerance, too. We found that these three genes are highly expressed together in the CA4 region of the hippocampal formation (Figure 8 and Supplementary Table S6).

Glucocorticoids can influence glutamatergic neurotransmissions at all relevant points, at the presynaptic transmitter release, at the postsynaptic receptor trafficking and function, and at transporter-mediated uptake and recycling of glutamate (Popoli et al., 2011). Therefore, we were interested in brain connectivity of circuits involved in emotional processing; we considered the FBN of the limbic circuit described above and co-localized it with the gene expression profiles of fkbp5, gmeb1, and gmeb2. High gene expression occurred in close vicinity to the hippocampal connectivity hub of the limbic lobe network (Figure 8). Distortions by gene polymorphisms active at a hub region may influence the integrity of the whole functional brain network.

### Connectome and Gene Expression Networks

Beyond the exploration of individual genes and connections, we used the knowledge model to reveal networks of brain structures with similar gene expression, structural and functional connectivity. We queried locations in the brain which were semantically related by a measured brain connectivity relation and a differential expression of genes relation. While the measured brain connectivity relation carries structural and functional connection strengths measured by DTI and rs-fMRI, respectively, the differential expression of genes relation is annotated by the number of differentially expressed genes between the related brain structures.

We used the average data sets from DTI “nki_dti_avg” and from rs-fMRI “nki_fc_avg” from the NKI Rockland study (Nooner et al., 2012) from the USC Multimodal Connectivity database (Brown et al., 2012) again. Differential expression of genes was computed based on the Allen Human Brain Atlas microarray gene expression data set. Values and brain regions were imported from supplementary material from the study Hawrylycz et al. (2012).

Several difficulties arose with this approach. Brain connectivity was calculated between MNI coordinates in the brain, differential expression of genes between brain structures. The NKI Rockland study used the Craddock200 atlas (Craddock et al., 2012) for parcellating the MNI 152 brain while the microarray gene expression data set used the Allen Human Brain Atlas ontology. We solved these issues by relating all MNI coordinates to their corresponding entries in the Craddock200 atlas in the knowledge model and, in turn, by manually mapping Craddock200 entries to corresponding entries in the Allen Human Brain Atlas ontology. We used the knowledge model to query all measured brain connectivity relations that had an origin and a target (in MNI coordinates) that ultimately relate to a context brain structure (which is mapped to the Allen Human Brain Atlas) that are origins and targets of a differential gene expression relation; however, this query returned no results.

**FIGURE 9:**
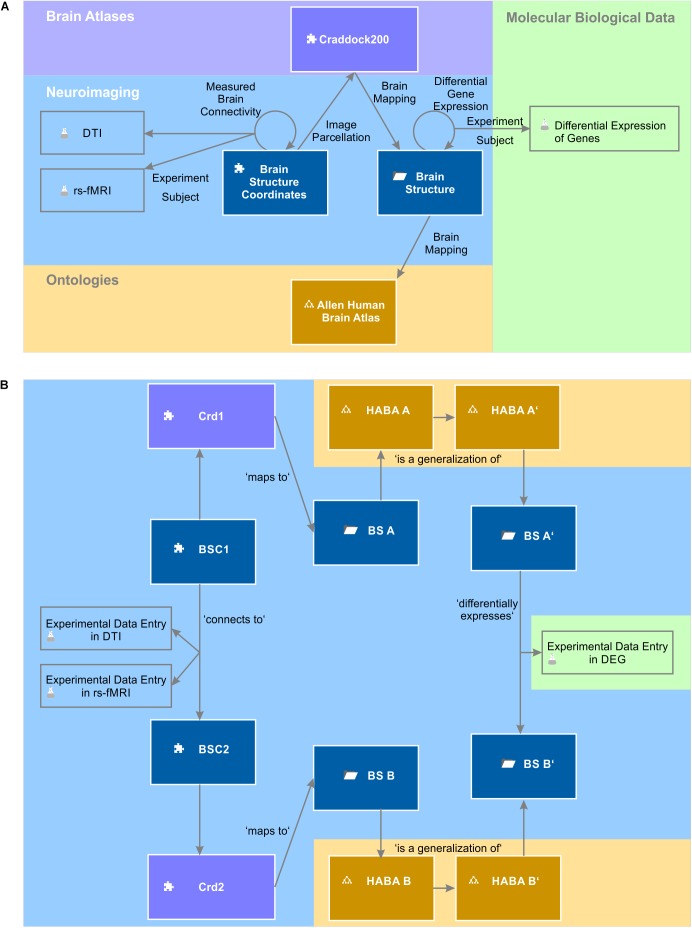
Knowledge model and instances of semantic objects and relations. **(A)** The query to relate gene expression networks with brain connectivity makes use of the depicted semantic objects and relations. A query describes a defined path through the semantic network. **(B)** The picture depicts the instantiation of the knowledge model described in **(A)**. The query returns experimental data entries in a differential expression of genes (DEG) experiment, which are indirectly related to experimental data entries in DTI and rs-fMRI experiments.

Gene expression experiments were related to finer brain structures than the gross-scale connectivity measurements. We therefore had to extend the query to find all contained sub-regions of associated areas in the Allen Human Brain Atlas. For this, we made use of the “is a” ontology relation and alternatively queried for all brain structures that are inferred from the related origin and target brain structures of the Measured Brain Connectivity relation in the Allen Human Brain Atlas. This query in fact returned 9571 hits (Supplementary Table S7). The part of the knowledge model which is used by this query is depicted in Figure 9. We filtered the results for all connections with a normalized structural connectivity of 0.1 and plotted normalized structural connectivity against functional connectivity and against differential gene expression. Additionally, we plotted a 3D-lattice graph of the obtained 607 structural, functional and gene expression connections (subset of Supplementary Table S7).

### Structural Connectivity Goes Along With Functional Connectivity and Homogeneous Gene Expression

**FIGURE 10:**
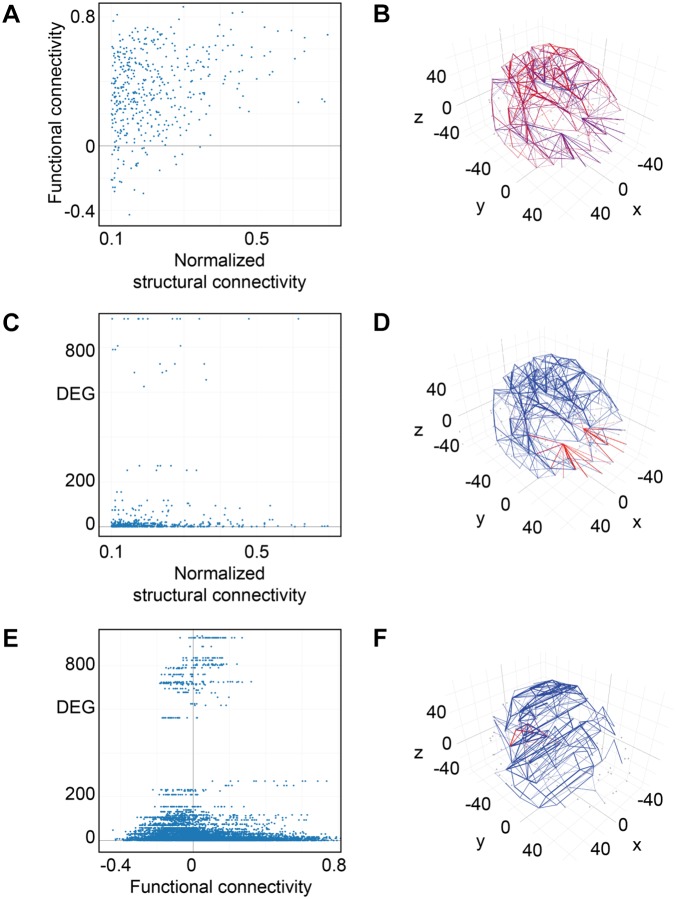
Connectivity vs. differential gene expression. **(A)** Normalized structural connectivity in terms of normalized fiber density from tractography (diffusion tensor imaging) is plotted against functional connectivity. Functional connectivity indicates the correlation in neural activity over time in the connected brain areas. Only those functional connections that also have a normalized structural connection strength of at least 0.1 were considered. **(B)** Structural and functional connectivity is depicted in a 3D-lattice graph. Structural connectivity is indicated by line thickness and functional connectivity by line color with red representing correlated neural activity (>=0.5), purple not correlated (<0.5) and blue negatively correlated neural activity (<0.0). **(C)** Normalized structural connectivity plotted against the number of differentially expressed genes (DEG). **(D)** Connections with an associated high differential expression of genes are surprisingly localized; all of them originate from the left and right putamen. Structural connectivity is indicated by line thickness and differential expression of genes by line color with red indicating high and blue low numbers of differentially expressed genes. **(E)** Functional connectivity is plotted against differential expression of genes (DEG). We only considered connections with a correlation value of neural activity of 0.5 or higher. **(F)** 3D-lattice graph of functional connectivity and differential gene expression. Line thickness indicates functional connectivity between values of 0.5 and 1.0 and line color indicates number of differential expression of genes with red high and blue low values. Those few connections with high differential gene expression (>200) and with functional connectivity (>=0.5) originate from occipital fusiform, lingual, parahippocampal, and Heschl’s gyrus.

For weak structural connections in terms of normalized fiber density, we found functional connections with positive correlation values in neuronal activity of the connected brain areas, others with no correlation as well as some with negative correlation values (Figure 10A). For a normalized structural connectivity greater than 0.4, we only obtained positive correlations in neuronal activity and there seems to be a trend toward higher functional connectivity for higher structural connectivity. Connections with high and low structural and functional connectivity were ubiquitously distributed over the entire brain (Figure 10B).

Interestingly, high numbers of differentially expressed genes, i.e., more than 100, appear for low normalized structural connectivity values only (Figure 10C). For normalized structural connectivity values greater than 0.4, the vast majority of associated brain structures had only a very low number of differentially expressed genes. There is a prominent spatial pattern of connections with highly differentially expressed genes though; we see highly differentially expressed genes for connections with a structural connectivity greater than 0.1 originating from the putamen and targeting in the forebrain and temporal lobe of both hemispheres excluding contralateral connections (Figure 10D and Table 1).

**Table 1:** Highly differentially expressed genes (DEG) between the putamen and structurally connected brain areas.

*Origin*	*Target*	*Normalized Structural Connectivity*	*Functional Connectivity*	*DEG Average*
*left putamen*	left parahippocampal posterior	0.163502106	0.020203011	687.5
*left putamen*	left planum temporale	0.18985185	-0.01262453	625
*left frontal pole*	left putamen	0.28089338	0.15076964	927
*left frontal pole*	left putamen	0.268176361	0.13167174	927
*left frontal medial*	left putamen	0.110934828	0.094022519	790
*left frontal pole*	left putamen	0.478702548	0.21312926	927
*left frontal pole*	left putamen	0.10229685	0.11504351	927
*left frontal orbital*	left putamen	0.355902746	0.32012312	726
*left frontal pole*	left putamen	0.200164537	0.1399255	927
*left frontal orbital*	left putamen	0.23406272	0.19123371	726
*left frontal pole*	left putamen	0.187038633	0.17440578	927
*right putamen*	right heschl’s	0.251257875	0.049333889	695
*right putamen*	right parahippocampal posterior	0.240377155	0.027959258	687.5
*right putamen*	right planum polare	0.361161921	0.1971855	655
*right frontal pole*	right putamen	0.135257294	0.2283248	927
*right frontal pole*	right putamen	0.342326336	0.16207665	927
*left frontal medial*	right putamen	0.103986254	0.14005883	790
*right frontal pole*	right putamen	0.174086567	0.13810447	927
*right frontal orbital*	right putamen	0.275009861	0.24960994	726
*right frontal pole*	right putamen	0.289418731	0.11346083	927
*right temporal pole*	right putamen	0.290537649	0.24174925	806.5
*right frontal pole*	right putamen	0.145893171	0.13850855	927
*right frontal pole*	right putamen	0.61517917	0.27000022	927
*right frontal pole*	right putamen	0.175577644	0.10383046	927
*right temporal pole*	right putamen	0.117579801	0.083963864	806.5


*Only connections with a normalized structural connectivity greater than 0.1 were considered. The table summarizes structural and functional connectivity and number of DEG averaged over all DEG values (Hawrylycz et al., 2012) between the connected brain areas.*

### Functional Connectivity Goes Along With More Homogeneous Gene Expression

Plotting all 9571 correlation values for neural activity against the number of differentially expressed genes revealed that most of the highly differentially expressed genes were measured for pairs of brain structures with weak or no correlation (Figure 10E). With increasing activity correlation values, numbers of differentially expressed genes decreased. The same trend is found for strong negative correlation values. Two brain areas can be considered functionality connected if their activity correlation over time is greater than 0.5. We found 362 relations between brain structures that have associated functional connectivity and differential gene expression measurements and visualized those connections by a 3D lattice model. Functional connections with homogeneous gene expression occur ubiquitously in the entire brain; however, a few functional connections with highly differentially expressed genes were localized to the occipital and temporal lobe (Figure 10F and Table 2).

**Table 2:** Strong connectivity and highly differentially expressed genes (DEG).

*Origin*	*Target*	*Normalized structural connectivity*	*Functional connectivity*	*DEG average*
*right lingual*	left occipital fusiform	0.264136489	0.73615919	271.6666667
*left lingual*	left occipital fusiform	0.22772869	0.60275624	271.6666667
*right lingual*	left parahippocampal posterior	0.143716534	0.50098513	251.5
*left lingual*	left parahippocampal posterior	0.30761773	0.55396672	251.5
*right lingual*	right occipital fusiform	0.177391911	0.51224682	271.6666667
*right lingual*	right occipital fusiform	0.235064117	0.68679426	271.6666667
*left lingual*	right occipital fusiform	0.02712589	0.61838067	271.6666667
*right lingual*	right parahippocampal posterior	0.33430536	0.51577434	251.5


*High DEG between the lingual gyrus and the occipital and temporal lobes. We found that these connections have a high functional connectivity (>0.5) and high DEG.*

We could identify nine pairs of brain structures which are structurally (>=0.5) and functionally connected (>=0.5) and have a low number of differentially expressed genes (<=10) (Table 3). There were no long-range connections among these.

**Table 3:** Strong connectivity and high similarity in gene expression.

*Origin*	*Target*	*Normalized structural connectivity*	*Functional connectivity*	*DEG average*
*left superior parietal lobule*	left superior parietal lobule	0.558669936	0.71112541	3
*left superior parietal lobule*	left superior parietal lobule	0.558669936	0.71112541	3
*right cingulate anterior*	right cingulate anterior	0.605500596	0.53997424	0
*right juxtapositional lobule*	right cingulate anterior	0.596213811	0.71527726	7.75
*right cingulate anterior*	right cingulate anterior	0.605500596	0.53997424	0
*right juxtapositional lobule*	right cingulate posterior	0.521552114	0.55794716	0.5
*right cingulate anterior*	right cingulate posterior	0.631735262	0.58602299	1.5
*right superior parietal lobule*	right superior parietal lobule	0.695758645	0.68872082	3
*right superior parietal lobule*	right superior parietal lobule	0.695758645	0.68872082	3


*Normalized structural connectivity (>=0.5) and functional connectivity (>=0.5) is high between areas of the parietal and cingulate cortex; these connections have, at the same time, a low DEG (<=10).*

## Discussion

We described a novel computational approach to transform a connectivity matrix into a semantic network. We used this approach to create a unified feature space for multimodal brain connectivity and gene expression data in which we created functional and structural connectivity and differential gene expression networks. We saw for all modalities that an increase in connectivity goes along with a more homogeneous gene expression pattern of the connected brain areas in the entire brain.

A recent study by Wang et al. (2015) describes a correspondence between resting-state activity and gene expression in cortical areas. This study further reported that specific genes are correlated with activity in the default mode network. A further link between cortical structure and gene expression was found by Romero-Garcia et al. (2018). They constructed structural covariance networks from MRI measures of cortical thickness and correlated those with gene expression measures from the Allen Human Brain Atlas (Hawrylycz et al., 2012) and affirmed the hypothesis that transcriptional networks and structural MRI connectomes are coupled. Both studies are in line with our observation that higher structural as well as functional networks go along with homogeneous gene expression. A further recent study found evidence for dissociable molecular signatures of limbic and somato/motor pathways in striatum (Anderson et al., 2018), which strengthens our observation that structural connections originating from the putamen show associated patterns of highly differentially expressed genes.

In an additional exploratory use case, we saw that the availability of modulators of the glucocorticoid receptors and of fkbp5, involved in stress hormone system regulation, are localized at a connection hub of the limbic lobe functional brain network. Fkbp5 was further discussed to have a role in schizophrenia (Binder, 2009). Schizophrenia is characterized as a dysconnection syndrome of fronto-temporo-limbic projections (Weinberger and Lipska, 1995). This circumstance points to the need to associate changes in brain connectivity in schizophrenia (Butz and Teuchert-Noodt, 2006; Bagorda et al., 2006) with genetic markers. Integrating connectome and genetic information in a semantic network can generate hypotheses that hold as a starting point for deeper analyses and assessments. Ideally one would co-localize gene expression data of fkbp5 with direct measurements of radiolabeled glucocorticoid receptor densities, for example, from PET imaging (Steiniger et al., 2008). Technically, the knowledge model would deal with receptor density data from PET in an analogous way as gene expression data measured in post-mortem brains.

Individual tools exist that link different sources of neuroscientific information, e.g., neuroimaging with gene expression data (Zaldivar and Krichmar, 2014; Rizzo et al., 2016); however, they are hard-coded to solve one particular aspect of neuroscience data integration. As soon as a new neuroscientific data source or brain ontology or atlas becomes available, software development is required to integrate them.

Semantic networks pose an ideal approach to integrate a highly multimodal and fast changing data space. They create a unified feature space in which facts being contained in high-dimensional and multi-modal brain data sets are explicitly represented and put into the permanently growing semantic context of neuroscience; with every new data source added, the contained knowledge in the knowledge model increases. The use of semantic networks solves two major problems. First, they homogenize data sets. Irrespective of their source and format, data sets become accessible to the query mechanism. Queries can combine any aspect of multiple modalities and report any desired combination of features, like structural and functional connectivity with gene expression. Secondly, the semantic network explicitly represents facts (e.g., which brain structures are connected), which are implicitly contained in tractography images or connectivity matrices (Rubinov and Sporns, 2010). Connected brain areas can then automatically be annotated by anatomical and functional meta-information from brain atlases and ontologies. Via the query mechanism, all this integrated knowledge becomes directly accessible to a user or can be exported in a machine-readable format for further processing (e.g., by novel connectomics algorithms combining connectivity and transcriptomic networks or by machine learning algorithms for automatized patient stratification).

Moreover, importing neuroimaging data into a semantic network ensures that imported data strictly adhere to a predefined standard. Storing methodological meta-information right from the planning stage of a study ensures reproducibility and comparability of data sets with future data sets and eases the exchange of data between different imaging centers. We extended the knowledge model by processing workflows in accordance to Alfaro-Almagro et al. (2018). A future task will be to extend the knowledge model toward common standards for planning and documenting brain imaging experiments such as the NeuroImaging Data Model (NIDM) (Keator et al., 2013; Maumet et al., 2016) for provenance modeling in human brain mapping. Integrating procedural information about processing workflows into the knowledge model creates a joined declarative representation of methods and data that can be queried from any desired perspective. The results are availability tables of fully documented patient assessments, which enable better comparison of neuroimaging results and increased interoperability.

Especially in a clinical setting, knowledge is of utmost importance for meeting the right treatment decision. All clinical fields dealing with the brain, however, face the tremendous challenge of managing an enormous amount of data for the individual patient and at the same time a rapidly changing scientific domain. For the individual clinician, keeping track of all new publications, tools, and knowledge bases, such as brain atlases, ontologies and web portals in neuroscience is nearly impossible. With the approach described here, it becomes possible to integrate knowledge about the connectome of the individual patient with the shared knowledge of the entire neuroscientific community. Ultimately, a unified knowledge model of brain data will enable a deeper understanding of brain diseases and more individualized treatment decisions in neurological and psychiatric therapy.

## Conclusion

Using semantic networks to create a neuroscientific knowledge base and applying it to connectome and neural receptor data enables the clinician to perform thorough patient stratification to compare the individual patient to all other patients seen before in an easily interpretable way. Further, it becomes possible to explore and assess neuroimaging data easily and straight-forward way and to relate the behavior of the patient to his brain pathology. By combining receptor and neuroimaging data, a better understanding of the disease state is achieved and planning individualized treatment becomes possible. Comparison of longitudinal brain scans quickly reveal whether the chosen treatment generates the desired effect in “repairing” the connectome of the individual patient.

## Author Contributions

SJK conducted the scientific study, assessed, presented, and discussed the results and contributed to the writing of the manuscript. MB-O invented the method, designed the study and contributed to the writing of the manuscript.

## Conflict of Interest Statement

MB-O and SJK are employed with Biomax and therefore will be affected by any commercial implications caused by this manuscript.

## Footnotes

1 http://www.knime.com

2 https://www.fmrib.ox.ac.uk/fsl

3 https://fsl.fmrib.ox.ac.uk/fsl/fslwiki/BET/UserGuide

4 http://surfer.nmr.mgh.harvard.edu/

## Supplementary Material

FIGURE S1 Semantic network of MRI T1 image processing workflow. We used a semantic network to represent the MRI T1 image processing as an example in the knowledge model. The element “Nifti image” is connected to the element patient and thereby connects the workflow to the patient data. FSL processing tools are indicated by rectangular objects with rounded corners; a rhombus indicates a linear transformation while a flag shape indicates a non-linear transformation. Colors are used to group different objects. Reddish colors indicate objects that hold image data in original coordinate space, while yellow indicates objects in MNI 152 coordinate space. Bluish colors were used for FSL calls and gray for transformations. Colors and shapes following Alfaro-Almagro et al. (2018).

TABLE S1 Mappings between different brain atlases. We manually created mappings for the 127 brain areas of the Craddock200 to other brain atlases and ontologies including PowerNeuron264, Human Allen Brain Atlas, FMA, FreeSurfer and Brede WOROI ontology.

TABLE S2 Functional brain network (FBN) of the limbic lobe. A query revealed 110 connections between brain areas that belong to the limbic lobe according to Allen Human Brain Atlas ontology classification which have associated DTI and resting state fMRI measurements from the NKI Rockland study.

TABLE S3 High gene expression of fkbp5. 211 measurements indicate the localized binding of probes to the fkbp5 gene in the brain. Only expression values greater than 8 and signals significantly greater than background noise (PACall value equal to 1) are listed.

TABLE S4 High gene expression of gmeb1. 79 measurements indicate the localized binding of probes to the gmeb1 gene in the brain. Only expression values greater than 8 and signals significantly greater than background noise (PACall value equal to 1) are listed.

TABLE S5 High gene expression of gmeb2. 11 measurements indicate the localized binding of probes to the gmeb2 gene in the brain. Only expression values greater than 8 and signals significantly greater than background noise (PACall value equal to 1) are listed.

TABLE S6 Brain area with high gene expression for fkbp5, gmeb1, gmeb2. The hippocampal CA4 region is the only area in which fkbp5, gmeb1 and gmeb2 is significantly highly expressed (expression value >= 8).

TABLE S7 Brain connectivity and transcription networks. A query revealed 9751 combinations of brain areas in which measurements of structural (DTI), functional connectivity (fMRI) and differential expression of genes (DEG) are available.
