# Supplementary figures and images for: From Matrices to Knowledge: Using Semantic Networks to Annotate the Connectome
Source: Front Neuroanat. 2018 Dec 7;12:111. doi: 10.3389/fnana.2018.00111 (PMC6292998; doi:10.3389/fnana.2018.00111)

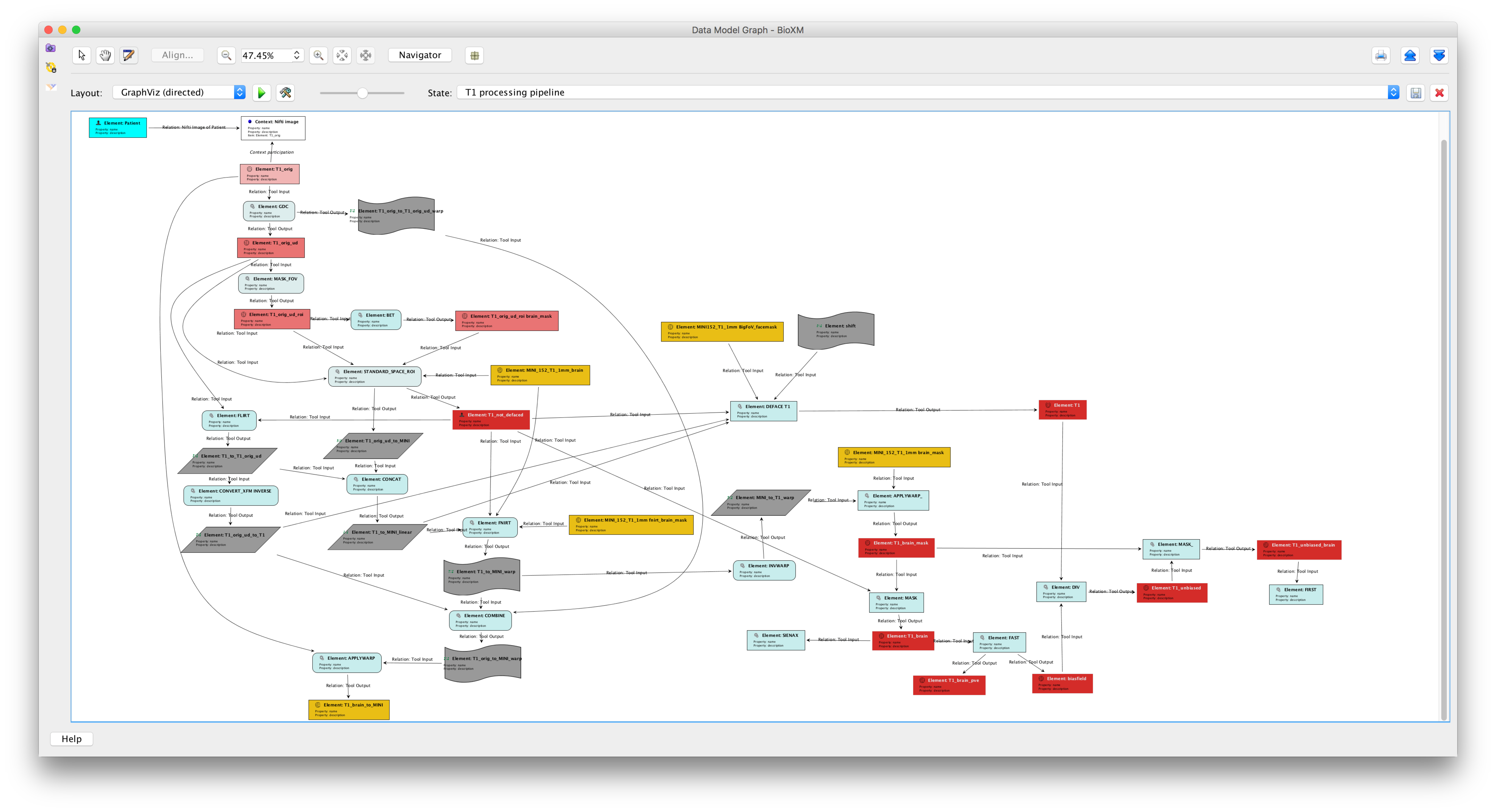

Supplement: FIGURE S1 — Semantic network of MRI T1 image processing workflow. We used a semantic network to represent the MRI T1 image processing as an example in the knowledge model. The element “Nifti image” is connected to the element patient and thereby connects the workflow to the patient data. FSL processing tools are indicated by rectangular objects with rounded corners; a rhombus indicates a linear transformation while a flag shape indicates a non-linear transformation. Colors are used to group different objects. Reddish colors indicate objects that hold image data in original coordinate space, while yellow indicates objects in MNI 152 coordinate space. Bluish colors were used for FSL calls and gray for transformations. Colors and shapes following Alfaro-Almagro et al. (2018). [file Image_1.TIF]
